# A Grid-Based Distributed Event Detection Scheme for Wireless Sensor Networks

**DOI:** 10.3390/s111110048

**Published:** 2011-10-25

**Authors:** Ja Won Ko, Yoon-Hwa Choi

**Affiliations:** Department of Computer Engineering, Hongik University, Seoul 121-791, Korea; E-Mail: kresource@naver.com

**Keywords:** grid-based sensor networks, fault detection, event detection

## Abstract

This paper presents a grid-based distributed event detection scheme for wireless sensor networks. The network is divided into square-shaped grids of predefined grid size, where sensor nodes in each grid form a cluster with a cluster head. Event detection at each grid alone based on the readings of its member nodes is limited in event detection performance, especially for a small event region compared to the grid size. To improve the performance, each grid is further divided into 2 × 2 sub-grids of equal size. The decision on an event is made by finding a square region of 2 × 2 sub-grids, not necessarily in the same grid, that passed a predefined threshold. This process is conducted at each cluster head in a distributed manner by inter-cluster communications. Event detection is initiated when a cluster head receives an alarm from its member nodes. The cluster-head communicates with its neighboring cluster heads to exchange the number of nodes reporting an alarm. The threshold for event detection can be dynamically adjusted to reflect the number of sensor nodes in a grid and event size, if known. High event detection accuracy is achieved with a relatively low threshold without sacrificing false alarm rate by filtering most errors due to transient faults and isolating nodes with permanent faults. Experimental results show that the proposed scheme can achieve high detection accuracy, while maintaining low false alarm rate.

## 1. Introduction

Wireless sensor networks, composed of a large number of small sensor nodes with sensing, computing, and wireless communication capabilities, often operate in an unattended mode to monitor various environments and detect events of interest [1]. Due to large-scale deployment of inexpensive sensor nodes, it is common for sensor nodes to exhibit faulty behavior. Hence it is important for a fault-prone sensor network to detect events in the face of fault-induced errors.

Several fault-tolerant event detection schemes have been proposed in [2–4]. Krishnamachari and Iyengar presented Bayesian algorithms to detect events in the presence of faulty sensor nodes [2]. They exploited the notion that measurement errors due to faults are likely to be uncorrelated, while measurements in a target region are spatially correlated. A fault-tolerant energy-efficient event detection scheme was proposed in [3]. For a given detection error bound, the number of neighboring nodes is determined to minimize the communication cost. Ding *et al*. [4] proposed a localized event boundary detection algorithm. Random bisection and trisection methods are employed to detect event boundary nodes. In [5] a secure event boundary detection scheme was presented to correctly identify event boundaries in adversarial environments. More recently, event detection using decision tree classifiers running on individual sensor nodes and applying a voting scheme to reach consensus among detections made by various sensor nodes has been proposed for disaster management [6].

Meanwhile, energy efficient data aggregation and routing in grid-based sensor networks have been investigated in [7–9]. In [7], a grid-based directed diffusion is presented. The network is divided into virtual grids and only one node in a grid-cluster participates in communication to reduce energy consumption. A clustering method based on virtual grid was proposed in [8]. Coordination mechanisms among heterogeneous nodes were also introduced. Yu *et al*. [9] proposed a grid-clustering routing protocol that provides scalable and efficient packet routing. A cluster grid construction scheme was presented to reduce energy consumption. In [10] an energy efficient framework for detecting events in sensor networks was presented. Clusters are used as local decision units. Cluster decisions are exchanged with one hop clusters that are likely to have been influenced by the event. An energy-efficient event notification scheme was also proposed. Event detection in grid-clustered sensor networks was investigated in [11]. Inter-cluster communications with some error corrections were used to improve event detection performance. A cellular approach to fault detection and recovery in sensor networks is presented in [12]. A virtual grid structure is used to detect energy-depleted nodes.

In wireless sensor networks, in general, due to a strong tradeoff between event detection accuracy and false alarm rate it is difficult to maintain high event detection accuracy for relatively small events or/and high fault probabilities, unless the tradeoff is greatly lessened.

In this paper, we present a grid-based, distributed, event detection scheme for wireless sensor networks, covering even relatively small event regions. To lessen the tradeoff the proposed scheme employs a smoothing filter to reduce the effect of transient faults. In addition, it maintains confidence levels of sensor nodes to isolate faulty nodes exhibiting errors for some extended period of time. Event detection locally at each grid based on the readings of its member nodes might achieve poor performance when an event region lies across multiple grids. To cope with this variations, a sensor network is divided into M × N square grids, each of which is further divided into 2 × 2 sub-grids. An event is detected by finding a square region of 2 × 2 sub-grids, not necessarily in the same grid, that passed a predefined threshold.

The rest of the paper is organized as follows. In Section 2, network structure, fault and event models are described. The proposed grid-based event detection scheme is presented in Section 3. Simulation results are shown in Section 4. Section 5 concludes the paper.

## 2. Network Structure and Fault/Event Model

In sensor network research, faults and events are often handled separately. Hence techniques for detecting faults in a wireless sensor network might not perform well as intended when both faults and events coexist in the network. Similarly, event detection techniques might not show the expected performance if fault behavior deviates from the predefined simplified model. In order to present our event detection scheme in the presence of various types of faults, we briefly describe our grid-based sensor network structure and fault/event model to be used throughout the paper. Grid-based sensor networks have been proposed for energy efficient data aggregation and routing. Our fault-tolerant event detection scheme is thus developed to conform to the basic protocol of the hierarchical networks.

### 2.1. Sensor Network Structure

**Figure 1.**
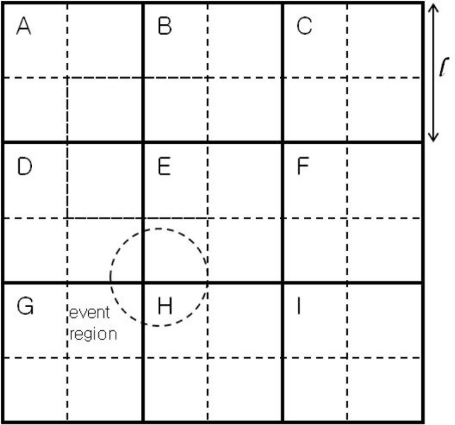
Sensor network structure for fault-tolerant event detection.

The sensor field is assumed to be divided into *M* × *N* square-shaped grids as illustrated in Figure 1, where there are nine grids, A through I, and *l* is the side of a square grid. Immediately after deployment, the sensor network is assumed to carry out grid construction process, and each sensor node figures out the grid it belongs to. Sensor nodes in each grid form a cluster, where a cluster head is selected dynamically. All other nodes in the cluster communicate directly with the cluster head, although multi-hop communication can be used without modification of the proposed event detection scheme. Two types of communication are defined here for event detection: one for communication between the cluster head and cluster members and the other for communication between neighboring cluster heads.

Although each grid can make a decision on an event based on the sensor readings of its member nodes, the accuracy might not be high especially for a relatively small event region. When such an event region lies across four neighboring grids, for example, each grid might have insufficient number of event-nodes to apply the well-known majority voting. In that case, high detection accuracy can only be obtained by lowering the threshold, resulting in a considerably high false alarm rate, except for low fault probability.

In order to cope with poor performance in the case of a small event region, we further divide each grid (in solid lines) into four sub-grids (in dotted lines) as shown in Figure 1, where each grid, except for the corners and sides, overlaps with eight square regions (SRs from here on) of 2 × 2 sub-grids, in eight different directions. In Figure 1, the grid E in the center, for example, has 8 overlapping SRs. In the NW direction, for example, there is an SR, in thick dotted lines. We name it SR*_ABDE_*, to indicate the four grids involved. In the N direction, the SR can be denoted by SR*_BE_* (*i.e.*, two sub-grids from grid B and two sub-grids from grid E).

An improved detection accuracy can be obtained if event detection is performed at each SR, along with the original grid. This extension requires inter-grid communications between neighboring cluster heads to send the information regarding the sensor readings at each sub-grid. As an illustration, the event region, in dotted circle in Figure 1, lies across the four grids D, E, G, and H. The event is most likely to be detected by a threshold test at SR*_DEGH_*.

The reason for using only 2 × 2 sub-grids is two-fold. First, any further divisions require additional memory and computation, and inter-grid communication overhead. Second, the resulting performance gains would be marginal unless a threshold test needs to be applied at a smaller sub-grid level.

### 2.2. Fault Model

Various types of faults may occur in sensor networks. Among others we focus on faults in sensor readings, due to malfunctioning sensors and noise. Some communication faults may also be covered as long as they can be modeled as faults in sensor readings.

Faults are assumed to occur in any nodes in the sensor network with the same probability. Each sensor node is assumed to know the range of normal readings. For clarity, we define “normal readings” to be the acceptable sensor data in the case of no-event. Any readings outside the normal range are named “unusual readings” for convenience. In other words, correct sensor readings in an event region are also called “unusual readings”. Hence each sensor node can make a binary decision on its own sensor reading, where a “1” indicates an unusual reading. Nodes in an event region will report a 1, although the range of sensor readings cannot be well defined, unless the nodes are faulty or some errors affect the correct readings.

Sensor readings of a faulty sensor node may lie in any range, including the normal and event ranges. Both permanent, transient, and intermittent faults are included in our fault model. Faults exhibiting errors for some extended periods of time may also be covered without modifying the proposed scheme. Transient faults are assumed to occur randomly and independently with the same probability. In the case of a permanent fault, both stuck-at-0 (normal) and stuck-at-1 (unusual) are assumed to occur with the same probability. In other words, sensor nodes with a stuck-at-0 fault always measure normal data, and they thus report a 0 even if they are in an event region.

### 2.3. Event Model

Fault-free sensor nodes in an event region are expected to measure some unusual values, reporting a 1 to the cluster head. In a fault-prone sensor network, however, incorrect reports due to faults are likely to occur, causing a false alarm. To cope with the false alarms while correctly detecting events, reducing the effect of faults along with a proper threshold is required. More specifically, the threshold needs to be sufficiently high to greatly reduce false alarm rate and low enough to achieve high event detection accuracy. In setting the threshold, the area of an event region plays an important role. For convenience we assume that an event region is a circle with radius *r*. Then the ratio of an event region area *A_ER_* to a grid area *A_G_* can be given by

(1) $$\frac{A_{\mathrm{ER}}}{A_{G}}=\frac{{\pi r}^{2}}{l^{2}}$$

For *l* = *mr*, the ratio $\frac{A_{\mathrm{ER}}}{A_{G}}$ for four different values of *m*, 1, 2, 3, and 4, are 3.14, 0.79, 0.35, and 0.2, respectively. In the case of *m* = 4, for example, at most 20% of the sensor nodes (in a grid) on average are in an event region, making it difficult to select a threshold value satisfying performance requirements. In a grid with *n* sensor nodes, the number of sensor nodes expected to be in an event region is $n\cdot \frac{A_{\mathrm{ER}}}{A_{G}}=\frac{n\pi }{m^{2}}$ on average. The numbers for various values of *n* and *m* are given in Table 1. If *n* = 15 and *m* = 4, for example, 3 nodes on average are expected to be in an event region, difficult to distinguish between events and faults as the fault probability increases.

For *n* ranging between 10 and 20, *m* needs to be less than 4 to have a few sensor nodes on average in an event region. In developing an event detection scheme, we will also take relatively small event regions into account to effectively adapt to varying network conditions.

## 3. Grid-Based Event Detection

Detecting events at each grid cluster alone in a grid-based wireless sensor network might be easy for a relatively large event region (e.g., *m* = 1). In that case, the majority of the sensor nodes in at least one grid are likely to report a 1, and thus voting schemes, such as the majority voting, can easily satisfy the requirements on detection accuracy and false alarm rate. For a relatively small event region (e.g., *m* ≥ 2), however, the number of sensor nodes reporting a 1 might be considerably small compared to the number of sensor nodes in a grid. Hence poor event detection performance might be unavoidable unless some measures are taken to significantly lower the threshold without increasing the false alarm rate.

As fault probability increases, the number of nodes reporting a 1 in the case of no-event also increases. Hence a voting scheme may have difficulty in distinguishing between faults and events using a threshold. The negative impact of faults can be greatly lessened by effectively reducing the fault probability. To realize it most of the erroneous readings due to transient faults are first corrected by employing a filter. In addition, nodes with permanent faults or reporting incorrectly for some extended periods of time are identified and isolated. Each cluster head maintains confidence levels of its member nodes indicating their records in reporting correctly. Sensor nodes with a permanent fault lose their confidence levels gradually, and they eventually reach the lower bound to be isolated from the rest. This fault management reduces the number of incorrect reports, allowing us to lower the threshold for event detection without sacrificing performance even for a relatively small event region. Two performance metrics, detection accuracy (DA) and false alarm rate (FAR), will be used in evaluating the effectiveness of the proposed scheme. DA is defined to be the ratio of the number of times that events are detected to the total number of event occurrences. FAR is defined as the ratio between the number of grids reporting an event, in the case of no event, and the total number of grids.

### 3.1. Reducing Erroneous Readings Due to Transient Faults

Transient faults may occur at any sensor nodes even though they are functional. Treating sensor nodes with transient faults as faulty nodes will reduce the number of usable sensor nodes, and it thus needs to be avoided. To effectively deal with transient faults, we employ a simple filter to correct most errors due to the faults. The reason for employing a filter is that an event will cause the sensor readings to be 1 for an extended period of time, while measurement errors due to transient faults might occur randomly and independently.

Let $x_{i}^{k}$ represent the binary sensor reading at time *t* = *k* at node *v_i_*. Then the filtered output $b_{i}^{k}$ is determined based on the *w* most recent readings with a threshold *q* as follows.

(2) $$b_{i}^{k}=1\;\;\;\;\;\text{if}\sum_{j=k-w+1}^{k}x_{i}^{j}\geq q$$

If *w* = 4 and *q* = 3, for example, $b_{i}^{k}$ can be 1 only if there are at least three 1’s out of four consecutive readings. This will correct most measurement errors due to transient faults unless they appear repeatedly for some extended period of time or at consecutive sampling times. Consequently, the number of false reports to the cluster head will be considerably reduced. The decision on an event at the cluster head will be made using a threshold test based on $b_{j}^{k}s$ from the member nodes. Due to the smoothing an event would be reported to the cluster head with some manageably small delay, depending on the window size *w* and *q*. The window size *w* depends on the sampling interval and the behavior of transient faults. In this paper, sampling period is assumed to be short, but long enough to treat transient faults independent. If some transient faults affect for an extended period of time such that the sensor readings are incorrect over several sampling times, they will be treated as they are. The window size needs to be small enough to minimize the delay involved. If necessary, however, an early warning can optionally be given to the cluster-head for its attention. Under the independence assumption the window size *w* and *q* are determined as follows.

Filtering out most errors induced by transient faults will effectively reduce the transient fault probability *p_t_*. For transient faults occurring randomly and independently in sensor nodes with the same probability *p_t_*, the effective transient fault probability *p̃_t_* for various values of *w* and *q* can be estimated using $\sum_{j=q}^{w}p_{t}^{j}{\left(1-p_{t}\right)}^{w-j}$, and the resulting reductions are shown in Table 2, where *p_t_* = 0.2 is assumed. If *w* = 4 and *q* = 3, for example, *p_t_* can be effectively reduced from 0.2 to 0.027.

The reduction in *p_t_* depends on the filter employed. The selection of a filter, however, might not be of importance as long as *p_t_* can be reduced in such a way that the tradeoff between detection accuracy and false alarm rate can be greatly lessened. As can be seen in simulation, the simple filter with *w* = 4 and *q* = 3 is good enough to achieve almost perfect performance even for *p_t_* = 0.2. The smoothing filter functions effectively for a wide range of *p_t_*, and can still function positively even when *p_t_* = 0.5. However, it might be reasonable to assume that *p_t_* is much smaller than 0.5 for wireless sensor networks to be used in environmental monitoring applications.

### 3.2. Isolating Faulty Nodes Using Confidence Level Evaluation

Permanent faults, unless the number of faulty nodes is negligibly small, also degrade the event detection performance at the cluster head. Since the number of faulty nodes is expected to increase with time, it is desirable to isolate them as soon as they are detected and identified. In our grid-based event detection, each cluster head receives reports from its member nodes, and makes a decision *D* based on a threshold *θ*, where *D* = 1 indicates an event. Each cluster head maintains confidence levels of its member nodes to isolate nodes with permanent faults when their confidence levels reach the assigned lower bound, resulting in better event detection performance at the cluster head. Depending on the decision made and the reports from its member nodes, the cluster head updates the confidence levels of the member nodes, reflecting the correctness of the reports. These updates need to be careful since fault-free nodes might generate some incorrect reports.

Let *c_k_*, ranging from 0 to 1 and initialized to 1, represent the confidence level of node *v_k_*. At the end of the event or fault detection cycle, the confidence levels of the member nodes are updated to reflect the correctness of their reports as follows.

For *D* = 0 (*i.e.*, the decision is no-event) in Table 3, the confidence level *c_k_* of node *v_k_* is increased by *α* to min (*c_k_* + *α*, 1) if the node reported a 0. If it reported a 1 instead, *c_k_* is lowered by *β* to max (*c_k_* − *β*, 0). The values of *α* and *β* need to be assigned depending on the fault behavior, if the best performance is necessary. In our evaluation, *α* = *β* = 0.1 is chosen without loss of generality.

For *D* = 1, on the other hand, it is not easy to figure out if *v_k_* reported correctly since the event boundary is unknown. Especially for a sensor node with limited resources and small event regions, it becomes difficult to locally figure out the exact boundary. As far as fault detection/isolation is concerned, however, it would be acceptable not to update the confidence levels when *D* = 1 for the following two reasons: (i) A stuck-at-1 node (outside the event region) can be identified and isolated when *D* = 0. Hence the last row in the table does not cause a problem; (ii) A stuck-at-0 node in an event region can hardly be identified in the case of no-event. It, however, can be detected if sensor readings of stuck-at-0 nodes do not change or are confined to a extremely small range while those of fault-free nodes vary notably over time. This type of stuck-at-0 can be identified and reflected in the confidence level even when *D* = 0, although we do not include this in the subsequent simulation in order to estimate the worst case performance of the proposed scheme.

If some existing sophisticated techniques are employed to figure out the exact boundaries, Table 3 can readily be modified. The simulation results in the next section, however, show that high performance can still be obtained even without isolating stuck-at-0 nodes when the permanent fault probability *p_p_* is 0.2. In addition, the performance gain achieved by removing stuck-at-0 nodes in that case will be shown to be marginal.

Sensor nodes are logically removed from the network and cannot participate in the event detection process when their confidence levels reach the lower bound (0 in this paper). Hence a sensor node *v_k_* with a permanent fault will gradually lose its confidence level *c_k_*, and then be isolated from the rest. On the other hand, if the isolation is due to transient or intermittent faults, the node can be reinstated when the behavior of the node changes later such that its confidence level reaches the upper bound (1 in the simulation).

### 3.3. Grid-Based Fault-Tolerant Event Detection

The proposed grid-based fault-tolerant event detection scheme consists of five steps. Initially each cluster head *H_i_* is assumed to know the numbers of sensor nodes in its four subgrids, $n_{i}^{0},n_{i}^{1},n_{i}^{2},n_{i}^{3}$. In addition, the four numbers of each of its neighboring grids are also assumed to be given. In fact, the numbers can be obtained right after deployment by intra- and inter-cluster communications. In Step 1, each sensor node *v_j_* computes $b_{j}^{k}$ based on *w* most recent readings. In Step 2, each sensor node with $b_{j}^{k}=1$ reports a 1 to the cluster head *H_i_*, and the cluster head counts the number of nodes reporting a 1 in each subgrid. Hence each cluster head will have the following eight numbers: $n_{i}^{0}$, $n_{i}^{1}$, $n_{i}^{2}$, $n_{i}^{3}$, $e_{i}^{0}$, $e_{i}^{1}$, $e_{i}^{2}$, $e_{i}^{3}$, where the first four represent the numbers of sensor nodes in the corresponding subgrids and the remaining four denote the numbers of nodes reporting a 1 in the four subgrids. The cluster head then computes the number of 1’s, *E*, in the grid. In the grid A, for example, $E_{A}=e_{i}^{0}+e_{i}^{1}+e_{i}^{2}+e_{i}^{3}$. The cluster head then applies a threshold test to determine on an event (*i.e.*, *D_A_* = 1 (*i.e.*, an event) if *E_A_* ≥ *θ*).

In Step 3, each cluster head receives the four numbers $e_{j}^{0},e_{j}^{1},e_{j}^{2},e_{j}^{3}$ from each of its neighboring cluster-head *H_j_*’s. It then computes the number of nodes reporting a 1 in each of the SRs (2 × 2 subgrids), and applies the same test with a threshold *θ* in Step 4. Finally in Step 5, depending on *D* and $b_{j}^{k}$ the confidence level of *v_j_* (*i.e.*, *c_j_*) is updated according to Table 3.

Due to the inherent tradeoff between DA and FAR, the value of *θ* is important to satisfy both requirements on DA and FAR. In determining *θ*, the number of nodes in a grid (or SR) and the event size are taken into account. We set the threshold *θ* at a grid to be

(3) $$\theta =\text{min}\;(\frac{d}{q_{d}},q_{c})$$

where *q_d_* and *q_c_* are predefined constants and *d* denotes the number of sensor nodes in the grid (or SR). For *m* = 3, in Table 1, about 1/3 of the nodes in a grid are in an event region on average. Hence to achieve high event detection accuracy for such a small event region, *q_d_* must be greater than 3. Under the assumption that *m* ≤ 3, we set *q_d_* to 4 to tolerate some variations due to nonuniform distribution of sensor nodes. Adjusting *q_d_* alone (i.e., $\theta =\frac{d}{q_{d}}$), depending on the event region size, might be good enough to achieve high performance. We, however, employ *q_c_* to achieve even higher DA with a negligibly small increase in FAR for relatively high *d*. The reason is that in a randomly deployed sensor network, very few sensor nodes might be placed in some sub-grids, even for high *d*. In that case, *q_c_* will allow the grid or SR to pass the threshold, while maintaining small FAR. If $\theta =\text{min}\left(\frac{d}{4},3\right)$, for example, the threshold *d*/4 is effective until it reaches 3. After that, it remains there.

Correcting most transient faults and isolating permanent faulty nodes allow us to lower *θ* to handle small event regions effectively. Since FAR is independent of the event region size, lowering *θ* will guarantee higher DA as event region size increases. If the event region size is approximately given based on experiments, adjusting the values of *q_d_* and *q_c_* accordingly will lead to a better performance.

Our proposed event detection scheme can be depicted as follows:

**Table:** 

Grid-based distributed event detection
Step 1. Given sensor reading $x_{i}^{k}$, each sensor node computes $b_{i}^{k}$. Step 2. Each sensor node with $b_{i}^{k}=1$ reports a 1 and each cluster head counts $e_{i}^{0}$, $e_{i}^{1}$, $e_{i}^{2}$, $e_{i}^{3}$, and apply a threshold test. Step 3. Obtain $e_{j}^{0}$, $e_{j}^{1}$, $e_{j}^{2}$, $e_{j}^{3}$ of each of its neighboring cluster-head *H_j_*. Step 4. Count the number of 1’s for each of the SRs and apply a threshold test with *θ*. Step 5. Update the confidence levels.

**Figure 2.**
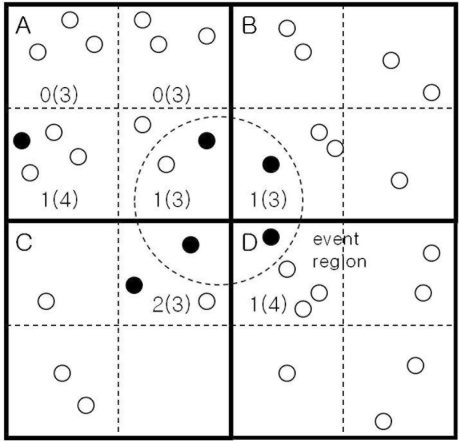
An illustration of the proposed grid-based event detection.

An illustration of the proposed event detection scheme is given in Figure 2, where there are only 4 grids, A through D. A small dotted circle in the center represents an event region placed across the four grids. The number within each subgrid, represents the number of sensor nodes (in the subgrid) reporting a 1. The total number of nodes in the subgrid is in the corresponding parenthesis. Among the four grids, grid A has only two 1’s out of 13 nodes. By performing inter-cluster (or grid) communications, the dotted SR (*i.e.*, SR*_ABCD_*) in the center can be found to have five 1’s out of 13 nodes, more likely to pass the threshold test.

Although we described that each cluster head applies the threshold test for eight SRs for convenience, in reality, it needs to apply the test to three SRs at most, in E, S, and SE directions. At the cluster head in grid A, for example, it needs to apply the test to SR*_AB_*, SR*_ABCD_*, and SR*_AC_*. The cluster head at grid B needs to apply the test only to the SR*_BD_*, and so on. Hence redundant threshold tests can easily be removed.

## 4. Simulation Results

Computer simulation is performed in a sensor network where 1,024 sensor nodes are randomly deployed in a 256 × 256 square area. The network is divided into 8 × 8 grids. Each grid is further divided into 2 × 2 sub-grids. Hence each grid has 16 nodes on average. Detection accuracy (DA) and false alarm rate (FAR) are employed as the performance metrics.

Experiments are conducted in the following order. First, we estimated the performance improvement due to the smoothing filter. We then evaluated the proposed grid-based event detection to show its effectiveness in achieving high performance even for small events. Finally, the effect of flattening *θ* for randomly deployed sensor networks is estimated.

**Figure 3.**
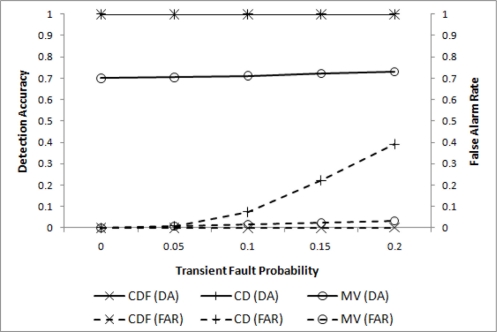
Improving DA and FAR using a smoothing filter.

In the first experiment, only transient faults are assumed to evaluate the effectiveness of filtering. Four different values of *p_t_* (0.0 ≤ *p_t_* ≤ 0.2) are chosen for *m* = 2 (*i.e.*, *l* = 2*r*), *w* = 4, *q* = 3, and $\theta =\text{min}\left(\frac{d}{4},3\right)$. The resulting DA and FAR are shown in Figure 3, where our CDF (cooperative decision with filtering) and CD (cooperative decision without filtering), and the well-known MV(majority voting) are compared.

For both CDF and CD almost perfect detection performance has been achieved, as expected. A significant difference, however, is noticed when FAR is compared. When *p_t_* = 0.2, FAR for CD reaches 0.4, while that for CDF remains very close to 0. MV, although inadequate for a small event region, shows relatively poor DA performance due to the insufficient number of sensor nodes to pass the threshold.

**Figure 4.**
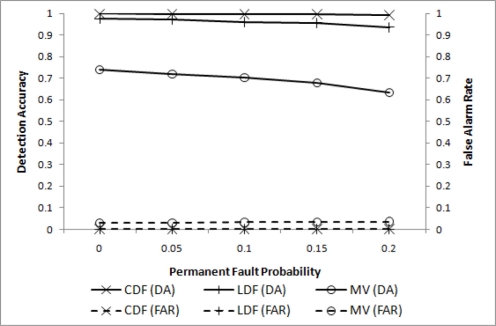
Comparison of CDF and LDF.

We then conducted simulation to evaluate the proposed grid-based event detection scheme. The performance is evaluated for four different values of *p_p_* when *p_t_* = 0.2. For comparison purposes, event detection without inter-cluster communications, named LDF (local decision with filtering), is also included. The performance of CDF is compared with LDF and MV in Figure 4, where effectiveness of the proposed scheme is demonstrated. Both DA and FAR for CDF are very close to 1 and 0, respectively, whereas LDF slowly loses its DA performance. For *p_p_* = 0.2, the difference is approximately 0.05. MV does not perform well as expected.

**Figure 5.**
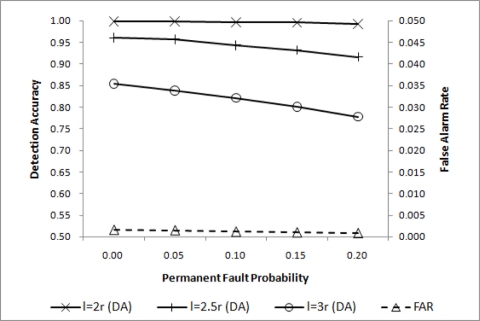
DA for $\theta =\text{min}\left(\frac{d}{4},3\right)$.

**Figure 6.**
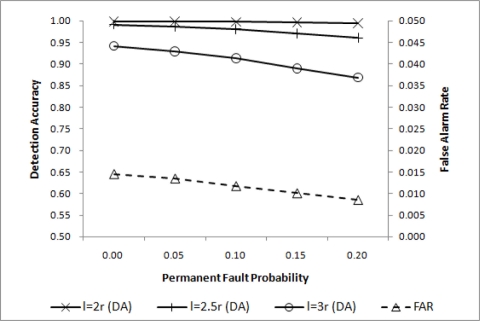
DA for $\theta =\text{min}\left(\frac{d}{4},2\right)$.

Event detection accuracy may change with the event size. In a relatively large event region, the simple majority voting will achieve high performance. As the event size becomes smaller, increasing *q_d_* is necessary to maintain high overall performance. DA for three different event region sizes are shown in Figure 5, where $\theta =\text{min}\left(\frac{d}{4},3\right)$ is shown to be adequate for *l* = 2*r*. For *l* = 2.5*r* and *l* = 3*r*, however, some improvements are desirable. Lowering *q_c_* will help improve DA with a negligibly small increase in FAR as shown in Figure 6, where $\theta =\text{min}\left(\frac{d}{4},2\right)$ is chosen.

**Figure 7.**
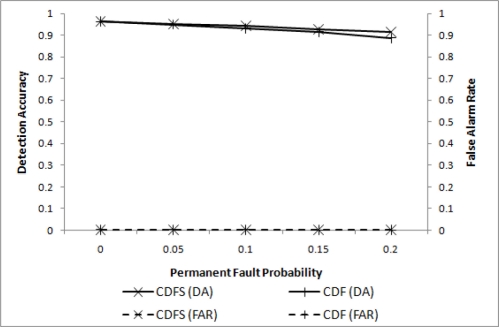
DA and FAR after isolating stuck-at-0’s for *p_t_* = 0.2, *w* = 4, *q* = 3, and *l* = 2.5*r*.

As addressed in the previous section, stuck-at-0 nodes can be detected when they are in an event region and the event boundary is identified. They can also be detected if sensor readings of a stuck-at-0 node are confined to a relatively small range over a long period of time compared to the readings of a normal node. If stuck-at-0 nodes are isolated, some additional gain can be expected. The improvements are shown as shown in Figure 7, where CDFS (cooperative decision with filtering and stuck-at-0-removal) and CDF (cooperative decision with filtering) are compared. As noted in the figure, the difference in DA is negligibly small for a relatively small *p_p_*.

Finally, we conducted simulation to see the performance changes due to flattening *θ*. DA and FAR for two different values of *θ*, $\theta_{1}=\frac{d}{4}$ and $\theta_{2}=\text{min}\left(\frac{d}{4},3\right)$, are shown in Table 4 Some notable improvements in DA are observed with a negligibly small increase in FAR. For *p_p_* = 0.2, DA improves by approximately 0.015 to reach 0.9922, whereas FAR increases only 0.00068.

## 5. Conclusions

In this paper, we presented a grid-based distributed event detection scheme for fault-prone wireless sensor networks. Sensor networks are divided into square grids to detect events locally with low communication overhead. To maintain high performance even with wide variations in node distribution and event size, each grid is further divided into 2 × 2 sub-grids. Events are then detected by finding a square region of 2 × 2 sub-grids that passed a predefined threshold. To reduce the impact of faults in decision making process, most false readings due to transient faults are smoothed out and sensor nodes with a permanent fault are isolated. Moreover, sensor nodes exhibiting incorrect readings for some extended period of time are temporarily isolated until they become stabilized. Computer simulation results have shown that a high DA can be achieved while maintaining an extremely low FAR for a wide range of fault probabilities, even for a relatively small event region.

## Figures and Tables

**Table 1.** $n\cdot \frac{A_{\mathrm{ER}}}{A_{G}}$ for various values of *m* and *n*.

***n***	***m* = 1**	**2**	**3**	**4**
10	31.4	7.9	3.5	2.0
15	47.1	11.85	5.25	3.0
20	62.8	15.8	7.0	4.0

**Table 2.** The effective transient fault probability *p̃_t_* for *p_t_* = 0.2.

***w***	***q* = 1**	***q* = 2**	***q* = 3**	***q* = 4**	***q* = 5**	***q* = 6**
2	0.360	0.040	-	-	-	-
3	0.488	0.104	0.008	-	-	-
4	0.590	0.181	0.027	0.002	-	-
5	0.672	0.263	0.058	0.007	0.000	-
6	0.738	0.345	0.099	0.017	0.002	0.000

**Table 3.** Updating *c_k_* at cluster heads.

***D***	***b_k_***	***c_k_***
0	0	min (*c_k_* + *α*, 1)
0	1	max (*c_k_* − *β*, 0)
1	0	no change
1	1	no change

**Table 4.** DA and FAR for two different threshold values *θ*_1_ and *θ*_2_ for *p_t_* = 0.2, *w* = 4, *q* = 3, and *l* = 2*r*.

***p**_p_*	**DA**		**FAR**	

	***θ*_1_**	***θ*_2_**	***θ*_1_**	***θ*_2_**
0.1	0.9900	0.9968	0.00021	0.00124
0.2	0.9770	0.9922	0.00024	0.00092
